# The Spectrum of Inborn Errors of Immunity in the United Arab Emirates: 5 Year Experience in a Tertiary Center

**DOI:** 10.3389/fimmu.2022.837243

**Published:** 2022-01-31

**Authors:** Hiba Mohammed Shendi, Amna Ali Al Kuwaiti, Ahmed Darwaish Al Dhaheri, Suleiman Al-Hammadi

**Affiliations:** ^1^ Department of Pediatrics, Tawam Hospital, Al-Ain, United Arab Emirates; ^2^ College of Medicine, Mohamed Bin Rashid University of Medicine and Health Sciences, Dubai, United Arab Emirates; ^3^ Department of Pediatrics, College of Medicine and Health Sciences, United Arab Emirates University, Al Ain, United Arab Emirates

**Keywords:** inborn errors of immunity, primary immunodeficiency, infections, genetic testing, therapy, mortality, United Arab Emirates

## Abstract

**Purpose:**

Inborn Errors of Immunity (IEI) are heterogeneous disorders of immunity with variable clinical presentation and outcome. This is the first comprehensive report from the United Arab Emirates aiming to describe the demographics, clinical characteristics, categories, treatment modalities and outcome of patients with IEI.

**Methods:**

This retrospective study was conducted on patients who attended Tawam Hospital between 2016-2020.

**Results:**

We identified 162 patients with IEI, of whom 152 were children. The age of onset of symptoms ranged between birth to 38 years. About two-thirds of patients were Emirati nationals, 64.2% had consanguineous parents and 38.3% of cases were familial. Patients were classified as; immunodeficiencies affecting cellular and humoral immunity (20.4%), combined immunodeficiencies with associated or syndromic features (38.3%), predominantly antibody deficiencies (16%), immune dysregulation (4.3%), congenital defects of phagocytes number or function (8.6%), defects in intrinsic and innate immunity (1.9%) autoinflammatory disorders (1.9%), complement deficiency (6.2%), bone marrow failure (1.9%) and phenocopies of inborn errors of immunity (0.6%). Genetic testing was performed in 85.2% of patients with a diagnostic yield of 92.7%. Complications included bronchiectasis, neoplasia, and vaccine-related infections. Immunoglobulin therapy and antimicrobial prophylaxis were both used in (51.9%) of patients while (20.4%) underwent hematopoietic stem cell transplantation (HSCT). The overall mortality rate was 10.5%.

**Conclusion:**

This report highlights the burden of IEI in the UAE. Ongoing education of physicians, establishment of a national registry and considering changes to early BCG vaccination are measures recommended to improve outcomes.

## Introduction

Human Inborn Errors of Immunity (IEI), previously known as Primary Immunodeficiency disorders are a group of heterogeneous disorders of immune function and regulation, affecting both children and adults. They are characterized by increased susceptibility to infection, predisposition to autoimmunity, autoinflammation, lymphoproliferation, granuloma formation, atopy and malignancy. In 2019, the International Union of Immunological Societies (IUIS) described 430 gene defects attributing to distinct disease phenotypes (1). By the end of 2020, twenty six genetic defects were added (2). IEI were long regarded as rare diseases, not often considered by physicians in the differential diagnosis (3). However, a recent expert report suggests that IEI are no longer rare, with an estimated collective worldwide prevalence of 1 in 10,000 to 1 in 50,000. The increase in prevalence is attributed to better definitions of clinical phenotypes as well as improved diagnostics; mainly the availability of next generation sequencing used in performing targeted gene panels, whole exome, and whole genome sequencing (1, 4–6). Based on the 2019 IUIS classification, IEI are classified into 10 groups: Immunodeficiencies affecting cellular and humoral immunity, combined immunodeficiency with associated or syndromic features, predominantly antibody deficiency, diseases of immune dysregulation, congenital defects of phagocytes, defects in intrinsic and innate immunity, autoinflammatory diseases, complement deficiency, bone marrow failure and phenocopies of PID (7). The clinical presentation varies between IEI phenotypes and reflects the role of the defective component in the immune system. Patients experience infectious and/or non-infectious manifestations with involvement of multiple organs, warranting a multidisciplinary approach for optimal management. Delay in diagnosis is common and is associated with increased morbidity (8). Full blood count and baseline immunological investigations, as well as referral to an immunologist should be performed as early as possible, when IEI is suspected. Molecular analysis is useful in establishing a definitive diagnosis, guiding specific therapies and genetic counselling (4–6). Treatment depends on the specific diagnosis and associated clinical manifestations and can provide disease control and/or complete cure. If left undiagnosed, IEI can result in chronic and serious complications, which are potentially fatal.

IEI have been described worldwide and in countries within the Middle East and North Africa (MENA) region, including Saudi Arabia, Oman, Kuwait, Qatar, Morocco, Tunisia, Turkey, Israel, Iran and Egypt (9). A recent report from the MENA registry highlighted the burden of IEI in the region and included 30 patients from the UAE (10). However, comprehensive, independent studies from the UAE on IEI are lacking and a national patient registry has not yet been established.

The UAE population in 2018 was estimated as 9,366,829 by the Federal Competitiveness and Statistics Authority of whom 67% were males and 33% females. Expatriates and immigrants account for up to 88% of the UAE population (11).

Tawam Hospital is a tertiary referral center and an affiliated hospital for the United Arab Emirates University. The Pediatric Allergy/Immunology service in Tawam Hospital is the only specialized Pediatric Immunology Service within the Emirate of Abu Dhabi to date, accepting referrals for mainly children but also some adults within Abu Dhabi and from the surrounding Emirates. The seed of this service started in 2004, and a full-time service was set in 2016. Patients are either referred to the out-patient clinic or for in-patient care if they have clinical manifestations requiring hospitalization. In this study, we aim to describe the Tawam Hospital experience in the diagnosis and management of IEI in the last 5 years (2016-2020). The main objectives include: (1) Description of the demographic and clinical characteristics of patients with IEI in the United Arab Emirates. (2) Classification of IEI in UAE according to the IUIS 2019 classification. (3) Determination of the morbidity and mortality associated with IEI in UAE.

## Methods

### Patients

A total of 753 patients were evaluated for IEI from January 2016-December 2020. Of those, 162 patients were diagnosed as having IEI; 152 were children (defined as younger than 16 years of age) and 10 were adults.

### Investigations

Immunological evaluation was performed through taking a thorough history, examination and laboratory evaluation. Depending on the clinical presentation, laboratory tests included all or some of the following; full blood count with differential, measurement of serum immunoglobulins (IgG, IgA, IgM and IgE), IgG subclasses, lymphocyte immunophenotyping (CD3, CD4, CD8, CD19 and CD16/56) and specific antibody titers to diphtheria, tetanus*, Haemophilus influenzae* and twelve pneumococcal serotypes. TRECS (T cell receptor excision circles) were measured on some cases when atypical severe combined immunodeficiency (SCID) was suspected. Dihydrorhodamine 1,2,3 (DHR 1,2,3) test to assess phagocyte function and functional complement assays CH50 and AH50 were performed, as indicated.

Whole exome sequencing was the most readily available molecular test; targeted gene panels, chromosomal microarray and whole genome were performed in some cases. These molecular tests were sent abroad while FISH testing was performed in-house. Some patients were evaluated in other centers and were referred after diagnosis for management and follow up.

### Classification

Patients were diagnosed based on IUIS 2019 classification (7). As per the European Society of Immunodeficiency working definition for clinical diagnosis of inborn errors of immunity, patients who fulfilled the clinical and laboratory criteria for a specific IEI classification group but a final diagnosis was not established, were regarded as having unclassified IEI and were included within their specific IEI classification group (12).

### Treatment

Treatment modalities varied according to the specific diagnosis and clinical presentation and included: prophylactic antimicrobials, immunoglobulin therapy, enzyme replacement, immunosuppressant agents including corticosteroids, biological and cytokine therapies, chemotherapy, hematopoietic stem cell transplantation (HSCT) and gene therapy. Hematopoietic stem cell transplantation and gene therapy were performed in specialized centers outside UAE because of lack of their availability within the country.

### Data Collection and Processing

Electronic medical records of patients who presented to the Allergy/Immunology service in Tawam Hospital from January 2016 to December 2020 were examined retrospectively to identify records tagged with relevant international classification of disease (ICD) codes for IEI or suspected IEI. Cases with an improbable IEI diagnosis following clinical and laboratory evaluation and cases with secondary immunodeficiency were excluded. All diagnosed IEI patients (n=162) were included and were advised to continue follow up at the clinic or day unit. A standard data collection form was used to gather demographic, clinical, laboratory, radiological and genetic information on patients. Data were anonymized and analysis was performed using Stata 16 (Stata Corp, College Station, Tx). Continuous variables were presented as means with standard deviations and medians with interquartile ranges (IQR). Categorical variables were presented as frequencies and percentages.

### Ethical Considerations

The study was approved by Tawam Hospital Research and Ethics Committee. Individual patient consent was not sought as this observational study involved secondary use of non-identifiable patient information, previously collected for routine patient care.

## Results

### Patient Characteristics

There was a total of 162 patients with IEI during the study period (85 males and 77 females). About two thirds (n=101, 62.4%) were Emirati nationals, while 37.6% were non-Emirati. Ninety-five patients (64.2%) were born to consanguineous parents while positive family history of IEI was reported in 62 patients (38.3%) (**Table 1**).

**Table 1:** Characteristics of 162 UAE patients with IEI.

Category	N (%)	Gender M/F	Nationality - Emirati	Consanguinity	Family history
N (%)	162 (100)	85/77	101 (62.4)	95 (64.2)	62 (38.3)
Immunodeficiencies affecting cellular & humoral immunity	33 (20.4)	19/14	19 (57.6)	26 (86.7)	18 (54.6)
Combined immunodeficiencies with associated or syndromic features	62 (38.3)	30/32	46 (74.2)	31 (52.5)	21 (33.9)
Predominantly antibody deficiencies	26 (16)	13/13	10 (38.5)	13 (59.1)	10 (38.5)
Diseases of immune dysregulation	7 (4.3)	5/2	4 (57.1)	6 (85.7)	0 (0)
Congenital defects of phagocyte number or function	14 (8.6)	8/6	10 (71.4)	10 (71.4)	5 (35.7)
Defects in Intrinsic and Innate immunity	3 (1.9)	1/2	2 (66.7)	3 (100)	0 (0)
Autoinflammatory disorders	3 (1.9)	2/1	2 (66.7)	0 (0)	0 (0)
Complement deficiencies	10 (6.2)	6/4	7 (70.0)	4 (57.1)	7 (70.0)
Bone marrow failure	3 (1.9)	1/2	1 (33.3)	2 (66.7)	1 (33.3)
Phenocopies of inborn errors of immunity	1 (0.6)	0/1	0 (0)	0 (0)	0 (0)

At the time of study, there were 152 children and 10 adults with IEI. Two of the adults were diagnosed with IEI in childhood. The median age of onset of symptoms was 5 months (IQR: 1-18 months). The median age at diagnosis was 24 months (IQR: 5.5-72 months). The median delay in diagnosis was 12 months (IQR: 2-48 months). The mean age of onset of symptoms was 21.8 months (0 months- 38 years). The mean age when an IEI diagnosis was made was 57.3 months (0 months - 43 years). The mean delay in diagnosis (defined as the time between the onset of symptoms to diagnosis) was 35.3 months (0 months-32 years).

### Distribution and Frequency of IEI

**Figure 1:**
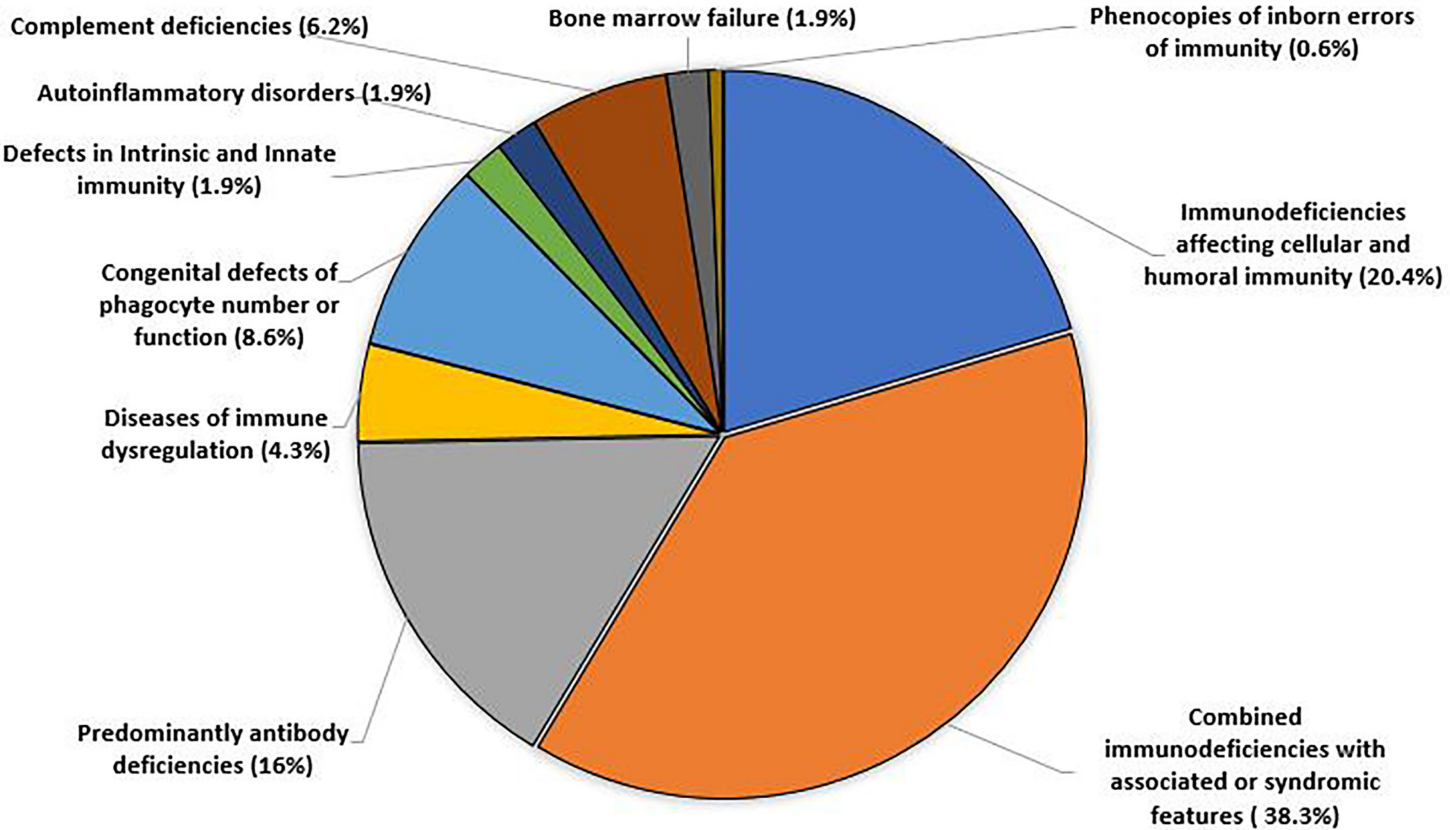
The percentages (distribution) of 162 patients according to IEI categories.

Patients with IEI were distributed according to IUIS 2019 classification into 10 different categories as shown in **Figure 1**.

The frequency of each IEI phenotype is shown in **Table 2**. Eight patients were regarded as undefined IEI, one with T-B+ severe combined immune deficiency (SCID), 2 with T-B- SCID, 4 with combined immunodeficiencies less profound than SCID and one with HLH. There was no clear predominance within the different forms of SCID or combined immunodeficiencies less profound than SCID (ranging between 1-4 cases in each group). Within the combined immunodeficiencies with associated or syndromic features, partial DiGeorge syndrome was the most reported syndrome in 31 patients. One patient was diagnosed simultaneously with DiGeorge syndrome and ataxia telangiectasia. Common variable immunodeficiency was the most frequent predominantly antibody deficiency, reported in 8 patients. Chronic granulomatous disease was the predominant congenital defect in phagocytes, reported in 9 patients. Of the complement deficiencies, hereditary angioedema was the most prevalent condition, observed in 8 patients.

**Table 2:** Frequency of each IEI in 162 UAE patients.

Category	N
**Immunodeficiencies affecting cellular and humoral immunity**	***33***
T-B+ severe combined immune deficiency (SCID)	*5*
* CD3D deficiency*	*1*
* IL2RG*	*1*
* JAK3 deficiency*	*2*
* Undefined*	*1*
T-B- SCID	*14*
* ADA deficiency*	*1*
* ARTEMIS deficiency*	*4*
* Cernounous (XLF) deficiency*	*3*
* RAG1 deficiency*	*3*
* Reticular dysgenesis*	*1*
* Undefined*	*2*
Combined immunodeficiencies less profound than SCID	*14*
* CD40 ligand deficiency*	*3*
* DOCK2 deficiency*	*1*
* DOCK8 deficiency*	*2*
* NIK deficiency*	*4*
* Undefined*	*4*
**Combined immunodeficiencies with associated or syndromic features**	***62***
Congenital thrombocytopenia	*1*
* Wiskott-Aldrich syndrome*	*1*
DNA repair defects	*16*
* Ataxia telangiectasia*	*14*
* Bloom syndrome*	*2*
DNA repair defects & Thymic defects with additional congenital anomalies	*1*
* Ataxia telangiectasia & DiGeorge syndrome*	*1*
Hyper-IgE syndromes (HIES)	*5*
* AD-HIES (Jobs)*	*3*
* Netherton syndrome*	*2*
* Cartilage hair hypoplasia*	*1*
Thymic defects with additional congenital anomalies	*31*
DiGeorge syndrome	*31*
Calcium channel defects	*1*
STIM1 deficiency	*1*
**Others**	***6***
* der(18)t(7:18)(q35;q22.2)*	*1*
* Hepatic veno-occlusive disease with immunodeficiency (VODI)*	*1*
* Kabuki syndrome*	*1*
* STAT5b deficiency*	*3*
**Predominantly antibody deficiencies**	***26***
*Severe reduction in all serum immunoglobulin isotypes with profoundly decreased or absent B cells, agammaglobulinemia*	*8*
* CD79 A Deficiency*	*2*
* X-linked agammaglobulinemia*	*5*
* ZNF699 deficiency*	*1*
*Severe reduction in at least 2 serum immunoglobulin isotypes with normal or low number of B cells, CVID phenotype*	*10*
* CVID with no gene defect specified*	*8*
* NFKB2 deficiency*	*1*
* PI3KR1 (APDS)*	*1*
Other antibody deficiencies	*8*
* Selective IgA deficiency*	*5*
* IgG 2 & IgG4 deficiency*	*1*
* IgG2 subclass deficiency*	*1*
* Transient hypogammaglobulinemia of infancy*	*1*
**Diseases of immune dysregulation**	***7***
HLH	*1*
* Undefined*	*1*
HLH susceptibility	*1*
* Hermansky Pudlak Syndrome Type 2*	*1*
Syndromes with Autoimmunity	*5*
* CD25 deficiency*	*1*
* LRBA deficiency*	*3*
* Prolidase deficiency*	*1*
**Congenital defects of phagocyte number or function**	***14***
Congenital neutropenia	*2*
Defects of motility	*3*
* Leukocyte adhesion deficiency type 3*	*3*
Defects of respiratory burst	*9*
* AR chronic granulomatous disease NCF1*	*8*
* X-linked chronic granulomatous disease, gp91phox*	*1*
**Defects in Intrinsic and Innate immunity**	***3***
Predisposition to Invasive Bacterial infections	*1*
* MyD88 deficiency*	*1*
Predominant susceptibility to viral infection	*1*
* STAT1 deficiency*	*1*
Others	*1*
* Osteopetrosis*	*1*
**Autoinflammatory disorders**	***3***
Recurrent inflammation	*3*
* Familial Mediterranean fever*	*3*
**Complement deficiencies**	***10***
Susceptibility recurrent pyogenic infections	*1*
* C3 deficiency*	*1*
Susceptibility to disseminated neisseria infections	*2*
* Factor D deficiency*	*2*
Others	*7*
* Hereditary angioedema type I*	*3*
* Hereditary angioedema type II*	*4*
**Bone marrow failure**	***3***
Dyskeratosis congenita	*3*
* RTEL1 deficiency*	*3*
**Phenocopies of inborn errors of immunity**	***1***
Associated with somatic mutations	*1*
* RAS-associated autoimmune leukoproliferative disease (RALD)*	*1*
*Total*	*162*

Bold and underline, to highlight IUIS classification.Italic, to highlight sub-categories

Clinical manifestations: The most prevalent clinical manifestations were infections, including: pneumonia, 106 cases (65.4%), otitis media, 53 cases (32.7%), sepsis, 38 cases (23.5%), abscess formation, 27 cases (16.7%) and sinusitis, 19 case (11.7%). Viral infections were reported in 61 patients (37.7%), whereas oral thrush and other fungal infections were reported in 38 (23.5%) and 21 patients (13%), respectively.

*BCG* infections secondary to vaccination occurred in 10 patients (6.1%). Only 2 patients (1.2%) suffered parasitic infections.

Non-infectious clinical manifestations included failure to thrive in 71 patients (43.8%), benign lymphoproliferation in 14 patients (8.6%), autoimmune thrombocytopenia in 11 patients (6.8%), autoimmune hemolytic anemia in 10 patients (6.2%), autoimmune endocrinopathy in 10 patients (6.2%), autoimmune enteropathy in 10 patients (6.2%) and arthritis in 2 (1.2%) patients. Atopic manifestations included asthma in 43 cases (26.5%), eczema in 31 cases (19.1%) and food allergy in 6 cases (3.7%) as seen in **Table 3**.

**Table 3:** Clinical manifestations of 162 UAE patients with IEI.

Infectious manifestations	N (%)
Pneumonia	106 (65.4)
Otitis media	53 (32.7)
Sinusitis	19 (11.7)
Abscess	27 (16.7)
Lymphadenitis	10 (6.2)
Meningitis	3 (1.9)
Osteomyelitis	1 (0.6)
Sepsis	38 (23.5)
Mycobacterial infection	10 (6.1)
Disseminated *Mycobacterial tuberculosis complex*	4 (2.5)
* Mycobacterial tuberculosis complex* adenitis	4 (2.5)
* Mycobacterial tuberculosis complex* brain abscess/tuberculoma	2 (1.2)
Viral infections	61 (37.7)
Oral thrush	38 (23.5)
Other fungal infections	21 (13.0)
Parasitic infection	2 (1.2)
Non-Infectious manifestations	**N (%)**
Immune dysregulation	
Benign lymphoproliferation	14 (8.6)
Autoimmune thrombocytopenia	11 (6.8)
Autoimmune hemolytic anemia	10 (6.2)
Autoimmune endocrinopathy	10 (6.2)
Insulin dependent diabetes mellitus	4 (2.5)
Autoimmune hypothyroidism	4 (2.5)
Autoimmune thyroiditis (asymptomatic)	2 (1.2)
Arthritis	2 (1.2)
Autoimmune enteropathy	10 (6.2)
Others	9 (5.6)
Exfoliative erythroderma	1 (0.6)
Vitiligo	1 (0.6)
Kawasaki disease	1 (0.6)
Alopecia areata	1 (0.6)
Cutanous granulomas	1 (0.6)
HLH	2(1.2)
Lymphoid interstitial pneumonia	1 (0.6)
Atopy	
Food Allergy	6 (3.7)
Eczema	31 (19.1)
Asthma	43 (26.5)
Failure to thrive	71 (43.8)

### Complications of IEI

Complications reported in our cohort were bronchiectasis in 24 cases (14.8%), neoplasia in 8 patients (5%) and vaccine-related infections in 13 patients (8%). Ten patients developed *Mycobacterial tuberculosis complex* infections following BCG, one patient developed measles and varicella following MMR vaccination, while Rotavirus gastroenteritis was reported in 2 patients following Rota virus immunization (**Table 4**).

**Table 4:** Complications of IEI in 162 UAE patients.

Complications	N (%)
Bronchiectasis	24 (14.8)
Neoplasia	8 (5.0)
EBV-positive Burkitt lymphoma	1 (0.6)
Large B cell lymphoma	2 (1.2)
ALL	1 (0.6)
MALT lymphoma in the lung	1 (0.6)
Atypical EBV-positive T-cell lymphoid proliferation of the bilateral larcrimal gland	1 (0.6)
Adenocarcinoma of stomach	1 (0.6)
Squamous cell carcinoma of the tongue	1 (0.6)
Vaccine related infections	13 (8)
Mycobacterial tuberculosis complex infection post BCG vaccine	10 (6.2)
Rota gastroenteritis	2 (1.2)
Measle infection	1 (0.6)*
Varicella infection	1 (0.6)*
Others	17 (10)
Pneumothorax	3 (1.9)
Lobectomy	2 (1.2)
Scarring in the lung	1 (0.6)
Hearing impairment	3 (1.9)
Blindness secondary to CMV retinitis	1 (0.6)
Acute kidney injury	1(0.6)
Malabsorption	3 (1.9)
Portal hypertension	1 (0.6)
Recurrent bleeding due to CMV colitis	1 (0.6)

*Same patient.

### Genetic Testing

Genetic testing was performed in 138 patients (85.2%) with a diagnostic yield of 92.7% (128 patients). One patient was diagnosed simultaneously with Di-George syndrome and ataxia telangiectasia. Seventy-eight (61%) of the IEI were inherited as autosomal recessive, while 40 were autosomal dominant/new mutations and 11 were X linked recessive conditions.

### Treatment

The most frequently used treatment modalities were immunoglobulin therapy and antimicrobial prophylaxis, both used in 84 patients (51.9%). Immunoglobulin therapy was used as replacement therapy in 76 patients and for immunomodulation in 8 patients. Thirteen patients (8.1%) received immunosuppressant therapy including corticosteroids, sirolimus, mycophenolate mofetil, cyclosporin and azathioprine. Biologics used included rituximab, anakinra, canakinumab, and adalimumab. Interferon gamma was used in one patient with CGD. Six patients with hereditary angioedema received enzyme replacement therapy in the form of C1 esterase inhibitor concentrate. Five out of 8 patients (62.5%) who developed neoplasia were treated with chemotherapy.

A total of 34 patients (21%) underwent hematopoietic stem cell transplantation (HSCT). Of those, 21 patients were diagnosed with immunodeficiencies affecting cellular and humoral immunity, 6 patients with congenital defects of phagocyte number or function, 2 patients with combined immunodeficiency with associated or syndromic features (one with Wiskott Aldrich syndrome and one with cartilage hair hypoplasia). One patient with predominantly antibody deficiency due to APDS received HSCT as well as 2 patients with diseases of immune dysregulation due to LRBA deficiency, one patient with CD25 deficiency and one patient with bone marrow failure. One patient with IL2R γ deficiency was treated with gene therapy (**Tables 5**, **6**). Of the 34 patients who received HSCT, 5 (14.7%) passed away.

**Table 5:** Frequency of therapies used in 162 UAE patients with IEI.

Management	N (%)
**Immunoglobulin therapy**	84 (51.9)
**Antimicrobial prophylaxis**	84 (51.9)
**Immunosuppressant therapy**	13 (8.1)
Corticosteroids	13 (8.0)
Sirolimus	3 (1.9)
Mycophenolate	3 (1.9)
Azathioprine	1 (0.6)
Cyclosporine	2 (1.2)
**Biologics/cytokines**	15 (9.3)
Rituximab	10 (6.2)
Anakinra	2 (1.2)
Canakinumab	1 (0.6)
Adalimumab	1 (0.6)
Interferon gamma	1 (0.6)
**Anti-inflammatory/Immunomodulators**	6 (3.7)
**Enzyme replacement** (C1 esterase inhibitor)	6 (3.7)
**Chemotherapy**	5 (3.1)
**Other**	3 (1.9)
G-CSF	1 (0.6)
Romiplostim	2 (1.2)
**Hematopoietic stem cell transplantation**	34 (21)
**Gene Therapy**	1 (0.6)

**Table 6:** Treatment modalities and mortality within IEI categories.

Classification	Immuno-globulin therapy	Anti-microbial prophylaxis	Immuno-suppressant therapy	Biologics cytokines	Anti-inflammatory/Immuno-modulators	Enzyme replacement	Chemo-therapy	Hematopoietic stem cell transplantation	Gene Therapy	Deaths
Overall, N=162	84 (51.9)	84 (51.9)	13 (8.1)	14 (8.6)	6 (3.7)	6 (3.7)	5 (3.1)	34 (21)	1 (0.6)	17 (10.5)
Immunodeficiencies affecting cellular & humoral immunity	32 (97.0)	29 (90.6)	1 (3.0)	5 (15.2)	0 (0.0)	0 (0.0)	2 (6.1)	21 (63.6)	1 (3.0)	7 (21.1)
Combined immunodeficiencies with associated or syndromic features	21 (33.9)	19 (30.7)	4 (6.6)	0 (0.0)	0 (0.0)	0 (0.0)	0 (0.0)	2 (3.2)	0 (0.0)	3 (4.8)
Predominantly antibody deficiencies	19 (73.1)	13 (50.0)	1 (3.8)	2(7.7)	1 (3.8)	0 (0.0)	1 (3.8)	1 (3.8)	0 (0.0)	1 (3.9)
Diseases of immune dysregulation	4 (57.1)	4 (57.1)	5 (71.4)	4 (57)	2 (28.6)	0 (0.0)	2 (28.6)	3 (42.8)	0 (0.0)	3 (42.8)
Congenital defects of phagocyte number or function	3 (21.4)	11 (78.6)	1 (7.1)	2 (14.3)	0 (0.0)	0 (0.0)	0 (0.0)	6 (42.9)	0 (0.0)	1 (7.1)
Defects in Intrinsic and Innate immunity	2 (66.7)	2 (66.7)	0 (0.0)	0 (0.0)	0 (0.0)	0 (0.0)	0 (0.0)	0 (0.0)	0 (0.0)	1 (33.3)
Autoinflammatory disorders	0 (0.0)	0 (0.0)	1 (33.3)	1 (33.3)	3 (100.0)	0 (0.0)	0 (0.0)	0 (0.0)	0 (0.0)	0 (0)
Complement deficiencies	0 (0.0)	2 (20.0)	0 (0.0)	0 (0.0)	0 (0.0)	6 (60.0)	0 (0.0)	0 (0.0)	0 (0.0)	0 (0)
Bone marrow failure	3 (100)	3 (100)	0 (0.0)	0 (0.0)	0 (0.0)	0 (0.0)	0 (0.0)	1 (33.3)	0 (0.0)	1 (33.3)
Phenocopies of inborn errors of immunity	0 (0.0)	1 (100)	0 (0.0)	0 (0.0)	0 (0.0)	0 (0.0)	0 (0.0)	0 (0.0)	0 (0.0)	0 (0)

### Mortality

Out of 162 patients with IEI, 17 died over the study period. The overall mortality rate was 10.5%. The highest mortality (42.8%) was in patients with immune dysregulation. This was followed by bone marrow failure and defects of intrinsic and innate immunity groups simultaneously, where one out of three patients (33%) died. The patient in the latter group was diagnosed with autosomal recessive STAT1 deficiency. The mortality within the subsequent groups was as follows: immunodeficiencies affecting cellular and humoral immunity (21.1%), congenital defects of phagocyte number and function (7.1%), combined immunodeficiencies with associated or syndromic features (4.8%) and predominantly antibody deficiencies (3.9%). No deaths were reported in patients with autoinflammatory disorders, complement deficiencies or phenocopies of inborn errors of immunity (**Table 6**).

## Discussion

IEI are a heterogeneous group of defects in immune function. This is the first comprehensive report from the UAE, describing 162 patients with IEI who presented to Tawam hospital over a 5-year period.

Although most of the UAE population are expatriates, around two thirds (62.4%) of IEI patients were Emirati. This can be attributed to the referral pattern, as government hospitals are main referral centers for Emirati citizens, as well as the high consanguinity rate within the Emirati population, which is estimated at 50.4% (13). In this population with IEI, the consanguinity rate was 64.2%, similar to that reported within the MENA region (60.5%) (10) and slightly lower than rates reported by neighboring Kuwait (78%) (14) and Oman (76%) (15), but significantly higher than Western counties such as the UK (2.9%) (16) and Germany (8%) (17).

There was positive family history of IEI in 62 patients (38.3%).

As expected in a pediatric immunology service, most patients were children. It is likely that this number is an under-estimate and does not reflect the true number of children in UAE with IEI. It is not possible to estimate the prevalence of IEI within the UAE, as data from other centers is lacking.

The mean age of onset of symptoms was 21.8 months (0 months- 38 years) and the mean delay in diagnosis was 35.3 months (0 months-32 years), comparable to that in the region (10). Delay in diagnosis is associated with increased morbidity and highlights the importance of raising awareness within physicians about IEI. A study conducted in UAE in 2012 to evaluate pediatrician awareness of the signs, symptoms and investigations of IEI suggested that further education was needed (18).

The most prevalent IEI was combined immunodeficiencies with associated or syndromic features (38.3%); due to partial DiGeorge syndrome which is the most common micro-deletion syndrome (19). It is clear that there is a high level of awareness among our pediatricians about the association of immunodeficiency with this syndrome. The second most common IEI category described in this population was immunodeficiencies affecting cellular and humoral immunity, followed by predominantly antibody deficiencies and congenital defects of phagocyte number and function, of which autosomal recessive CGD was reported in all except one case.

A similar distribution was observed by our colleagues from neighboring Kuwait, except that immunodeficiencies affecting cellular and humoral immunity were more prevalent than combined immunodeficiencies with associated or syndromic features (14). Although predominantly antibody deficiencies are the most common IEI reported in European registries (16, 17, 20) and within the MENA region (10), only 16% of our cohort were affected. CVID is the most prevalent IEI within this group and it is mainly diagnosed in adults. The low number of adults in this cohort, and a milder phenotype of predominantly antibody deficiencies may have contributed to under-reporting of this group. Autoinflammatory disorders and bone marrow failure syndromes were also under-represented, likely because they are mainly managed by rheumatologists and hematologists, respectively.

Whereas, the reported infections are long recognized manifestations of IEI, we also observed a range of non-infectious manifestations and complications including failure to thrive, benign lymphoproliferation, autoimmune cytopenia, autoimmune endocrinopathy, autoimmune enteropathy, arthritis, neoplasia, atopy and others, emphasizing the crucial role of the immune system in self-tolerance, immune-regulation and tumor immunity.

Infectious complications included bronchiectasis in 14.8% of cases and vaccine related infections in 13 patients, of whom 10 (6.2%) developed serious *Mycobacterial tuberculosis* infections post BCG vaccination. The risk of developing potentially fatal, disseminated BCG following vaccination in patients with IEI, has been reported in the UAE and other countries where the BCG vaccine is administered shortly after birth (21–24). Including SCID newborn screening within the national program, is proven to provide early diagnosis for immunodeficiencies affecting cellular and humoral immunity, allowing avoidance of administration of live vaccines early in life and improving outcomes. Delaying the BCG vaccine to 6 months of age has been suggested (22) and has recently been implemented in Saudi Arabia.

Genetic testing was performed in a higher percentage (85.2%) compared to other countries within the MENA region (34.3%) (10), including Kuwait (69%^)^ (14), and Qatar (36.6%) (25). The diagnostic yield was high (92.7%). For those with a positive result, screening of family members, and genetic counselling were offered. Most IEI were inherited in an autosomal recessive manner, similar to reports from other MENA countries (9, 10, 14).

Treatment modalities included immunoglobulin therapy, antimicrobial prophylaxis and HSCT, similar to other reports from the region (10, 14, 15, 25). We also reported the use of immunosuppressants, biologics, and enzyme replacement.

The mortality rate was 10.5%, higher than that reported by European countries such as the UK (6.3%) and Italy (4.2%). This can be attributed to the higher prevalence of more severe forms of IEI that are inherited as autosomal recessive, in our study. Heightened awareness of inherited immunodeficiencies in Europe and the US, with earlier diagnoses, may also explain better outcomes. However, the mortality rate described in our cohort was lower than that reported in within in the MENA region (15.8%) (10), including other gulf countries such as Qatar (21.4%) (25), Oman (18%) (15) and Kuwait (26%) (14). Given the evolving nature of IEI, follow up is warranted for ongoing management and assessment of long term morbidity and mortality.

In the UAE, the availability of genetic testing early during the process of immunological evaluation, assisted in providing more accurate and specific diagnoses and was helpful in guiding definitive therapy.

## Conclusion

This study is the first to fully describe IEI in the UAE. Our findings are comparable to other reports from the Gulf region. However there are more prominent non-infectious manifestations and complications, and lower mortality. There is a higher prevalence of more severe forms of IEI and higher mortality rate compared to European countries. Genetic testing is a clinically useful tool in guiding specific diagnosis and management. Ongoing education of physicians on IEI is required to avoid diagnostic delay. The establishment of a national registry with collaboration of immunology centers in the country is required to evaluate the prevalence and full impact of IEI within the UAE.

## Data Availability Statement

The data generated for this study are included in this manuscript. Any further inquiries can be directed to the authors.

## Ethics Statement

This study was reviewed and approved by Tawam Hospital Research and Ethics Committee. Written informed consent from the participants’ legal guardian/ next of kin was not required to participate in this study in accordance with the institutional requirements.

## Author Contributions

HS: Patient diagnosis, data collection, analysis & interpretation, writing & design of manuscript. AAK: Data collection, analysis & interpretation, review of manuscript. ADD: Data collection, analysis & interpretation, review of manuscript. SA: Patient diagnosis, review of manuscript. All authors have read and approved the final manuscript and agree to be accountable for the content of the work.

## Conflict of Interest

The authors declare that the research was conducted in the absence of any commercial or financial relationships that could be construed as a potential conflict of interest.

## Publisher’s Note

All claims expressed in this article are solely those of the authors and do not necessarily represent those of their affiliated organizations, or those of the publisher, the editors and the reviewers. Any product that may be evaluated in this article, or claim that may be made by its manufacturer, is not guaranteed or endorsed by the publisher.
